# Supplementary material for: Distinct roles of brain network flexibility in motor learning across age
Source: Imaging Neurosci (Camb). 2026 Sep 9;4:IMAG.a.1359. doi: 10.1162/IMAG.a.1359 (PMC13559766; doi:10.1162/IMAG.a.1359)
Supplement: Supplementary file 1 [file IMAG.a.1359_supp.pdf]

Supplementary Material

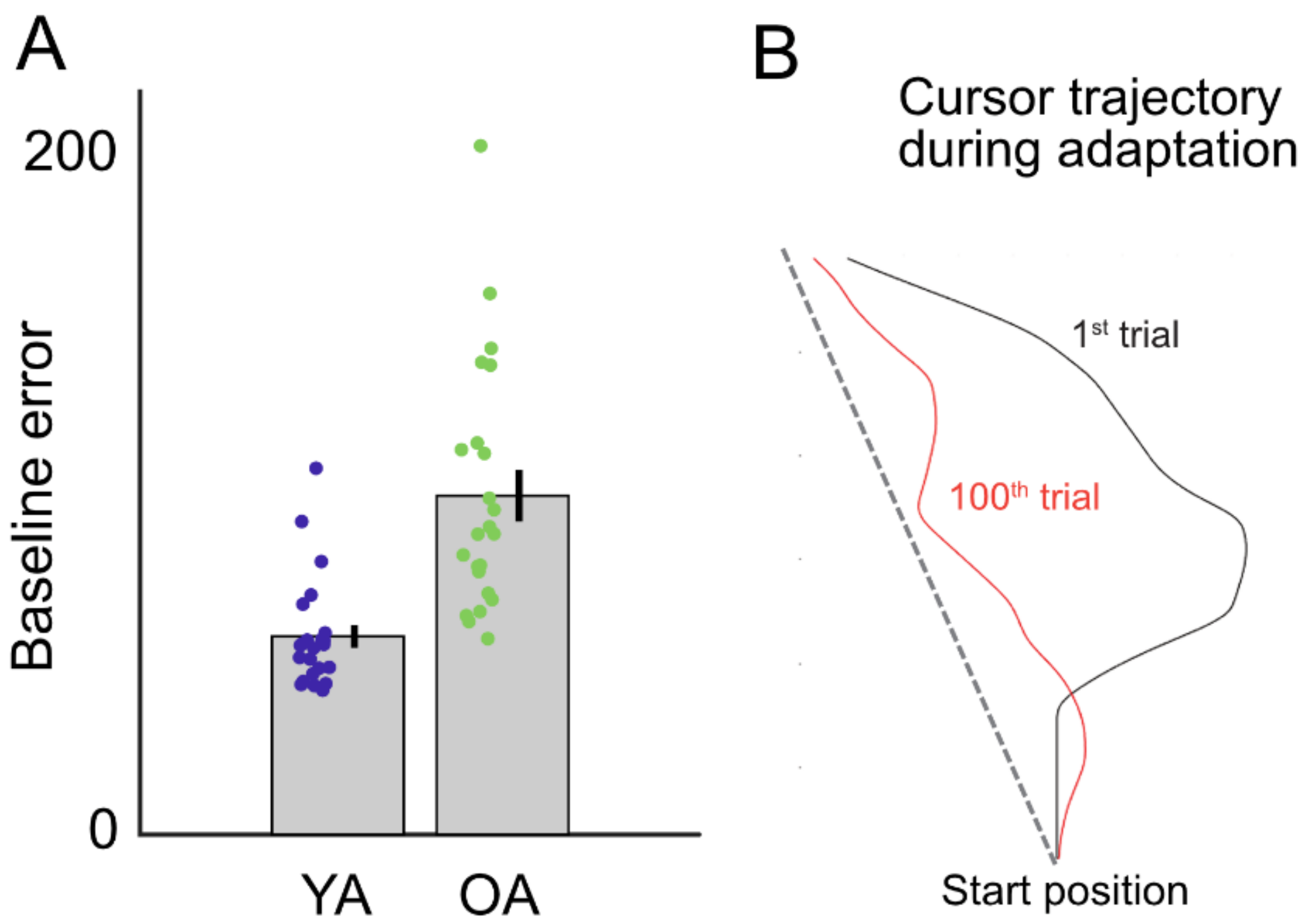

Supplementary Figure 1: Baseline motor performance **(A)** and a typical cursor trajectory during adaptation **(B)**. **A**. Group-averaged error at baseline across 50 trials. Each dot represents data from an individual participant, and each error bar indicates the standard error of the mean across participants. **B**. A typical cursor trajectory during adaptation from a representative participant in the YA group. The cursor deviation decreased consistently across the 100 trials.
